# Magnetically Treated Water in *Phaseolus vulgaris* L.: An Alternative to Develop Organic Farming in Cuba

**DOI:** 10.3390/plants12020340

**Published:** 2023-01-11

**Authors:** Yilan Fung Boix, Albys Ferrer Dubois, Yanaisy Perez Quintero, Elizabeth Isaac Alemán, Cristiane Pimentel Victório, Jorge González Aguilera, Malgreter Noguera Betancourt, Luis Morales-Aranibar

**Affiliations:** 1 National Center for Applied Electromagnetism, Santiago de Cuba 90600, Cuba; 2 Faculdade de Ciências Biológicas e da Saúde, Universidade do Estado do Rio de Janeiro, Campus Zona Oeste—UERJ-ZO, Rio de Janeiro 23070-200, Brazil; 3 Pantanal Editora, Rua Abaete, 83, Sala B, Centro, Nova Xavantina 78690-000, Brazil; 4 The Office of Innovation, Technology Transfer and Intellectual Property at the National Intercultural University of Quillabamba, Universidad Nacional Intercultural de Quillabamba, Cusco 08741, Peru

**Keywords:** common bean, magnetic field, photosynthesis, physiological parameters, seeds

## Abstract

*Phaseolus vulgaris* L. (common bean) significantly contributes to the human diet due to its protein, vitamin and mineral contents, making it one of the major edible plant species worldwide. Currently, the genetic resources conserved in germplasm banks in Cuba have experienced a loss of viability, which makes their propagation difficult. Magnetically treated water has been used to improve the response of seeds and plants of different species. However, there is little experimental evidence on the cultivation of the common bean irrigated with magnetically treated water or its positive effects on seed germination recovery and its effects on physiological, anatomical and morphological characteristics. This study aims to evaluate the growth and development of common bean with magnetically treated water as an alternative to rejuvenate the seeds for organic agriculture. A two-group experimental design was used: a group of plants irrigated with water without a magnetic field and a group of plants irrigated with water treated with a magnetic field at induction in the range of 100 to 150 mT. There was an increase of 25% in the percentage of germination; the stomatal anatomical structures behaved normally; and the stem length, vigor index, leaf area and seed weight increased by 35, 100, 109 and 16%, respectively. The concentrations of chlorophyll a, chlorophyll b pigments and carbohydrates in the plants grown with magnetically treated water were also stimulated in relation to control plants with increments of 13, 21 and 26%, respectively. The technology employed in this study did not have negative effects on the plant nor did it affect the presence of structures or the net content of the assessed compounds. Its use in the cultivation of *Phaseolus vulgaris* L. might represent a viable alternative for the improvement of the plant in organic farming production.

## 1. Introduction

*Phaseolus vulgaris* L. (common bean) belongs to the group of leguminous plants with edible seeds containing a high nutritional value. Beans are grown on all five continents and are an essential staple food, especially in Central and South America [1]. The common bean belongs to the Leguminosae family, which comprises 727 genera and approximately 19,000 species. Of these species, only five belonging to the genus Phaseolus are cultivated worldwide to form part of human food (*P. vulgaris* L., *P. coccineus* L., *P. acutifolius* A. Gray, *P. lunatus* L. and *P. polyanthus* Greenman) [2,3].

*Phaseolus vulgaris* L. is preferred within the bean species for economic and scientific purposes [4]. The bean is native to America, and its domestication has taken place in northern Andean and Mesoamerican populations as two gene pools [2,5]. The common bean was brought to Europe as an ornamental plant, and over time, it began to be cultivated in almost all parts of the world [2,6]. Common bean is an early maturing crop with a reasonable degree of drought tolerance, which has led it to play an essential role in farmers’ strategies in drought-prone lowland regions [7].

In Cuba, the consumption of common bean is very popular due to its high caloric content, which includes, among other elements, phosphorus, vitamins and iron. There are more than 20 improved bean varieties and cultivars in the country [8,9]. Crop yields are 9723 and 110,765 kg ha^−1^ for state and nonstate agricultural sectors, respectively, which cannot meet demand [10]. Over the past decade, the nonstate agricultural sector, mainly consisting of farms and small plots with very diverse conditions and low availability of agrochemical and energetic inputs, was mostly in charge of bean production in our country [11,12]. Many of these productions are mainly based on organic agriculture.

The organic agricultural production system is promoted as a low-cost ecological production system that improves human and soil health, improves the environment and increases agricultural sustainability [13]. Organic agriculture includes cultivation by small, low-income farmers. These low-input productions, if properly managed, can avoid susceptibility to water stress and low soil fertility and avoid storage pests, which are the main constraints to the yield of most crops [14]. In Cuba, the country’s conditions have forced the more extensive use of organic agriculture as a way of achieving higher quality production with lower inputs, and beans have been one of those crops produced on this ecological basis.

For better crop yields of *Phaseolus vulgaris* L. and to meet the Cuban population’s demand, new technologies that would allow large-scale bean production with the use of environmentally friendly techniques and better seed quality are needed. The success of agricultural production initially depends on the quality of the seeds planted. The term “seed quality” refers to the ability of the seed to germinate and have the vigor to develop in the new growing environment [15]. It is well-known that seed germination and viability can vary greatly from year to year and from one production site to another, as well as depending on how they are conserved [15,16,17]. Currently, advances in seed technology that stimulate growth and high vigor in the seedling phase have been obtained using films with silver nanoparticles on watermelon seeds (*Citrullus lanatus* L.) [18]; bioplastics consisting of modified starch and chitin mixed with *Bacillus subtilis*, used as a coating for corn seeds [19]; and pelleting with cellulose, diatomaceous earth and soy protein in broccoli seeds (*Brassica oleracea* L.) [20], among other techniques, such as the use of magnetically treated water [21].

One feasible technology is the application of magnetic fields to irrigation water in agricultural, industrial and household irrigation systems with the use of high-performance permanent magnet devices. In Cuba, specifically in Santiago de Cuba province, GREMAG^®®^ technology was employed for the magnetic treatment of irrigation water [21]. This technology has been used in different plant species, such as medicinal plants [22], ornamental plants [23] and vegetable plants [24], with satisfactory results.

This technology improves plant growth variables and nutrient uptake [24,25,26,27,28]. This type of treatment has been used to promote the best performance of several crops, such as *Solanum lycopersicum* L. [24,29,30,31,32], *Capsicum annuum* L. [33,34], *Cucumis sativus* L. [35], *Vicia faba* L. [36], *Beta vulgaris* [37], *Raphanus sativus* [38], *Cicer arietinum* L. [39] and *Phaseolus vulgaris* L. [40,41,42]. However, there is little experimental evidence on the cultivation of *Phaseolus vulgaris* L. irrigated with magnetically treated water or its positive effects on seed germination recovery and its effects on physiological, anatomical and morphological characteristics.

The objective of this paper is to assess the growth and development of *Phaseolus vulgaris* L. sown with magnetically treated water, which may improve the development and production of high-quality beans for human consumption.

## 2. Results

### 2.1. Determination of the Effect of Irrigation with Magnetically Treated Water on the Germination of Phaseolus vulgaris L.

**Figure 1:**
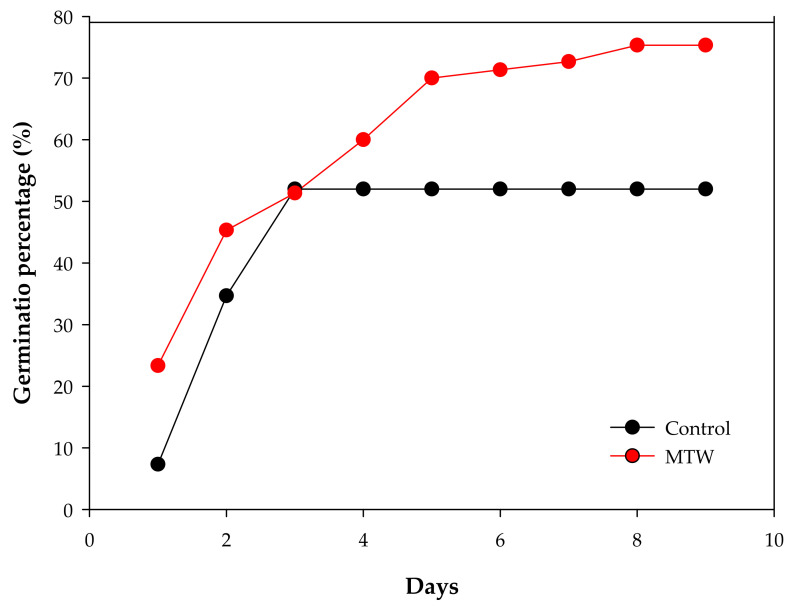
Germination percentage of *Phaseolus vulgaris* L. grown with magnetically treated water (*p* < 0.05). Control: plants grown without magnetically treated water, MTW: plants grown with magnetically treated water under a static magnetic field between 100 and 160 mT.

The germination percentage showed statistically marked differences between the mean values of the two experimental groups with a confidence level of 95.0%. Plants grown with magnetically treated water showed 75% germination compared to 50% germination in control plants (Figure 1). The difference between treatments represents a gain of 25% in favor of the magnetic treatment of irrigation water (Figure 1).

Similar results were reported for seed germination rates for both experimental groups from day 1 to 2. From day 3 to 8, the germination percentage increased in the plant group treated with magnetically treated water, while it stayed constant in the control group. This suggests that magnetic field application had a positive impact on seed germination and that it can be effective from the onset of the sowing of *Phaseolus vulgaris* L., regardless of the species variety.

Mean values of the hypocotyl length and vigor index in *Phaseolus vulgaris* L. plants treated with magnetized water were markedly different compared to the control plant group, with a 95% probability level (Table 1). The vigor index values suggest statistically significant differences (*p* < 0.05) between the two experimental groups. The length of the hypocotyl also increased within the same range; these data are of great importance for the assessment of the vigor index.

### 2.2. Determination of the Effect of Irrigation with Magnetically Treated Water on the Anatomical and Morphological Characters of Phaseolus vulgaris L.

**Figure 2:**
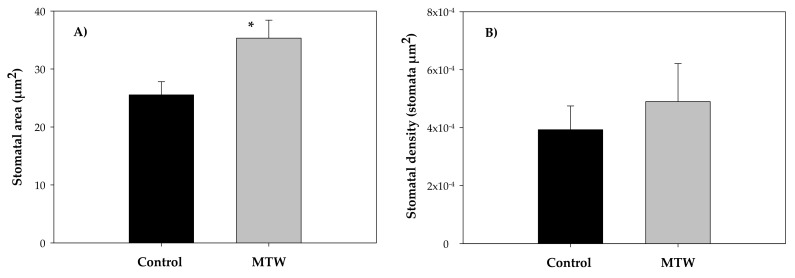
Mean values of stomatal area (**A**) and stomatal density (**B**) in *Phaseolus vulgaris* L. plants grown with magnetically treated water. * indicates statistically significant differences (*p* < 0.05). Control: plants grown without magnetically treated water, MTW: plants grown with magnetically treated water under a static magnetic field between 100 and 160 mT.

**Figure 3:**
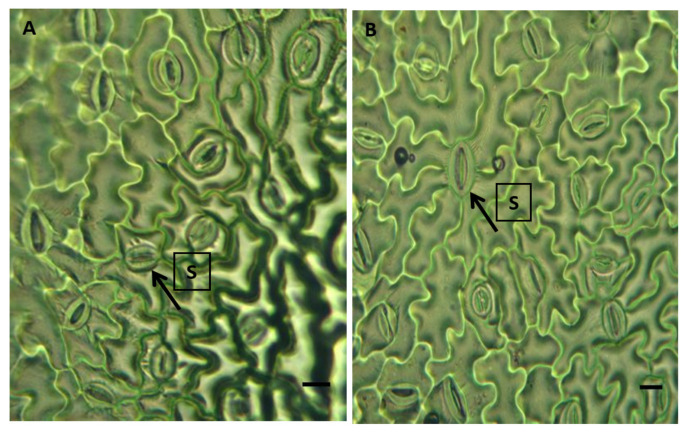
Microphotograph of stomata (S) in a leaf of *Phaseolus vulgaris* L. (**A**) Plants grown without magnetically treated water (control). (**B**) Plants grown with magnetically treated water (MTW). A 40× magnification. Bright field optical microscopy.

The stomatal area estimates showed statistically significant values of 35.32 ± 3.11 µm^2^ for plants treated with MTW compared to control plants (25.54 ± 2.27 µm^2^) (Figure 2A). Despite the fact that stomatal density was not significantly different between the two experimental groups (Figure 3), from a biological point of view, it was greater for plants grown with MTW (4.9 × 10^−4^ ± 1.31 stomata µm^2^) compared with the control (3.93 × 10^−4^ ± 0.82 stomata µm^2^) (Figure 2B).

Magnetically treated water under an induction range of 100 to 150 mT increased water absorption and enhanced the transpiration process. It was confirmed that the stomal structural characteristics of *Phaseolus vulgaris* L. grown with MTW remained unchanged (Figure 3). This study demonstrated that magnetically treated water increased absorption and transpiration processes in *Phaseolus vulgaris* L.

**Figure 4:**
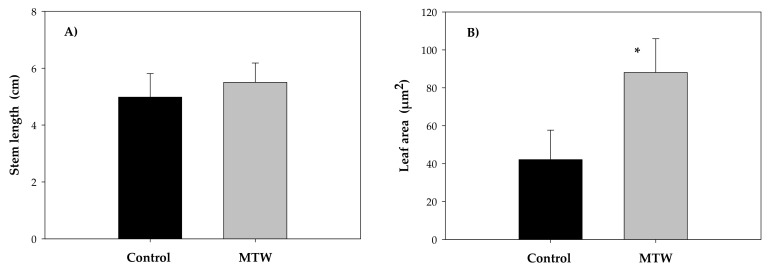
Mean values of stem length (**A**) and leaf area (**B**) in *Phaseolus vulgaris* L. grown with magnetically treated water. * indicates statistically significant differences (*p* < 0.05). Control: plants grown without magnetically treated water, MTW: plants grown with magnetically treated water under a static magnetic field between 100 and 160 mT.

The mean values of the stem length in *Phaseolus vulgaris* L. did not show statistically significant differences (Figure 4A). From a biological standpoint, however, the higher values correspond to the plant group irrigated with MTW (5.50 ± 0.68 cm) compared to the control group (4.98 ± 0.83 cm). In contrast, the leaf area values showed statistically significant differences, with a significance level of 95%. The leaf area value was 88.12 ± 17.83 µm^2^ for the treated group and 42.12 ± 15.51 µm^2^ for the control group (Figure 4B).

**Figure 5:**
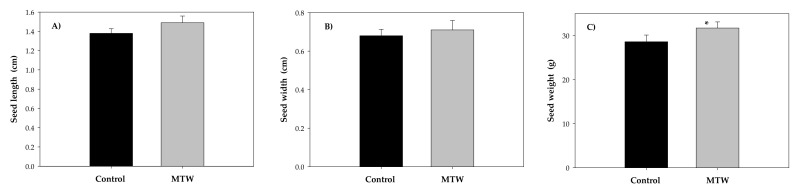
Mean values of seed length (**A**), seed width (**B**) and seed weight (**C**) in *Phaseolus vulgaris* L. grown with magnetically treated water. * indicates statistically significant differences (*p* < 0.05). Control: plants grown without magnetically treated water, MTW: plants grown with magnetically treated water under a static magnetic field between 100 and 160 mT.

Magnetically treated water impacts seed length, width and weight (Figure 5). Seed weight was the variable that showed statistically significant differences with respect to the control, with a significance level of 95% (Figure 5C). The treated group showed the highest seed weight values (31.73 ± 1.39 g) compared to the control plants (28.62 ± 1.51 g). Although there were no statistically significant differences (*p* < 0.05) in seed length (Figure 5A), the group of plants grown with magnetically treated water exhibited higher values (1.494 ± 0.070 cm) than the control plants (1.382 ± 0.047 cm). Similar results were obtained for the seed width, where magnetically treated plants were 0.71 ± 0.049 cm wide, and seeds in the control group were 0.68 ± 0.034 cm wide (Figure 5B).

In general, the results obtained in this experiment show that magnetically treated water had a positive impact on the anatomical and morphological features associated with the productivity of *Phaseolus vulgaris* L. seeds treated with an induction range of 100 to 150 mT.

### 2.3. Determination of the Effect of Irrigation with Magnetically Treated Water on the Physiological Characteristics of Phaseolus vulgaris L.

**Figure 6:**
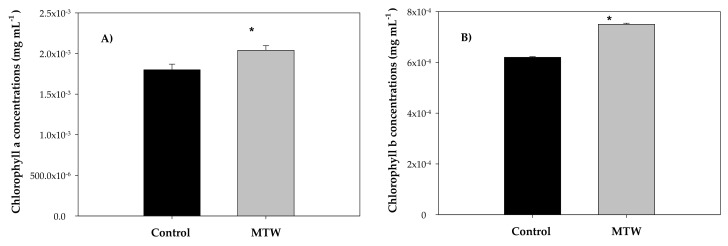
Mean values of chlorophyll concentrations in *Phaseolus vulgaris* L. grown with magnetically treated water. * indicates statistically significant differences (*p* < 0.05). Control: plants grown without magnetically treated water, MTW: plants grown with magnetically treated water under a static magnetic field between 100 and 160 mT.

Chlorophyll pigments were determined for *Phaseolus vulgaris* L. species, in which chlorophyll a absorbs violet, blue, orange—reddish and red wavelengths of radiation and a few intermediate wavelengths of radiation (green–yellow–orange) (Figure 6). Accessory pigments include chlorophyll b and carotenoids, which act as antennas, conducting the energy they absorb toward the reaction center. Chlorophyll molecules are found in the reaction center and can transfer their excitation as useful energy in biosynthetic reactions.

The plants grown with MTW showed higher chlorophyll a content than the control group, with values of 2.04 × 10^−3^ ± 5.74 × 10^−5^ mg mL^−1^ and 1.8 × 10^−3^ ± 6.9 × 10^−5^ mg mL^−1^, respectively. Statistically significant differences, with a significance level of 95%, were found for the mean values of chlorophyll a and b content in both experimental groups. The chlorophyll b content values in plants irrigated with magnetically treated water and the control group plants were 7.5 × 10^−4^ ± 4.6 × 10^−5^ mg mL^−1^ and 6.2 × 10^−4^ ± 2.5 × 10^−5^ mg mL^−1^, respectively.

**Figure 7:**
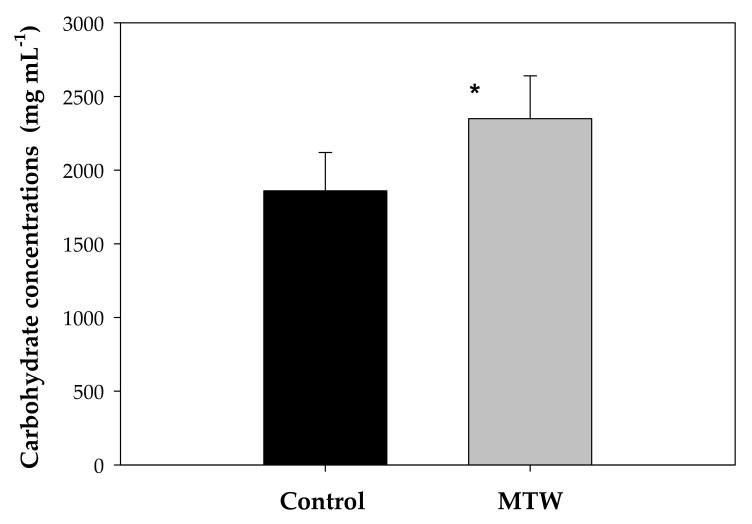
Carbohydrate concentrations in *Phaseolus vulgaris* L. grown with magnetically treated water * indicates statistically significant differences (*p* < 0.05). Control: plants grown without magnetically treated water, MTW: plants grown with magnetically treated water under a static magnetic field between 100 and 160 mT.

Figure 7 presents the mean values of the carbohydrate concentration in the experimental groups of *Phaseolus vulgaris* L., where the MTW group exhibited higher values (2350 ± 290 mgmL^−1^) compared to the control group (1860 ± 260 mg mL^−1^). Statistically significant differences with a probability of *p* < 0.05 were observed.

## 3. Discussion

The results obtained agree with the findings of Torres et al. [29], who reported a higher germination percentage for *Oryza sativa* L. (rice) and *Solanum lycopersicum* L. (tomato) after using magnetic fields of 10 and 15 mT. In the studies conducted by Grewal and Maheshwari [43] with *Pisum sativum* L. (pea) and *Cicer arietinum* L. (chickpea), magnetically treated water produced a significant increase (*p* < 0.05) in the germination percentage under an induction range between 3.5 and 136 mT. Selim et al. [27] also found that magnetically treated water increased the vigor index of *Solanum lycopersicum* L. (tomato), *Triticum aestivum* L. (chickpea) and *Pisum sativum* L. (pea) seedlings by 18.3, 81.9 and 65.1%, respectively, when compared to control plants.

The impact of magnetic field application on the germination process of plant species varies according to the magnetic inductions used. Plants grow under the effect of treated water that has different characteristics owing to changes in the water molecule structure, hydrogen bridges, pH variations and electrical conductivity [26,38]. These distinctive features of irrigation water favor the physiological and metabolic processes that occur during the germination of *Phaseolus vulgaris* L. from hypocotyl elongation to leaf appearance. This explains why the germination percentage and vigor index obtained in the group of plants grown with MTW were higher than those of control plants. Mroczek-Zdyrska et al. [42] found that the stimulation of plants by a weak permanent magnetic field (130 mT) increased the mitotic activity in the meristematic cells of the common bean. There was no influence of the 130 mT magnetic field stimulation on the development of aboveground plant parts.

In both experimental groups, stomata exhibited normal anatomical features, with two occlusive cells and three adjacent epidermal cells (Figure 3). Epidermal tissue has structures that are made up of joined cells, including stomata, formed by an ostiole, surrounded by two kidney-shaped occlusive cells which may or may not be accompanied by other epidermal cells that are structurally and physiologically associated with the stomata [44]. Cell wall thickening was observed in the inside rather than the outside. This structural adaptation of stomata confers on them the elasticity that enables them to open or close stomatal pores depending on turgid cell pressure [37].

Stomatal area values indicate that plants grown with magnetically treated water showed a larger stomatal aperture, which is proportional to an increased transpiration process. Certain environmental conditions, such as high relative humidity, have an impact on plant growth and can affect stomatal area and density. Although this may cause higher plant water consumption, a balanced use of water can be attained. As *Phaseolus vulgaris* L. shows a better water absorption rate, allowing its optimization, hydric equilibrium and plant growth are enhanced. Cultivation of this plant species is possible under extreme environmental conditions. Similar stem length values have been reported by Ahamed et al. [33] for *Capsicum annuum* L. (sweet pepper) seeds irrigated with magnetically treated water with an induction of 57–60 mT.

The lack of differences in stem length may be associated with meteorological conditions and the sowing time of *Phaseolus vulgaris* L. Khattab et al. [41] did not find significant differences in the stem length of *Phaseolus vulgaris* L. when compared with two varieties (Goya and Hama) of the species; however, this variable did show a significant increase due to the influence of weather conditions when varieties of the species were sown at different times of the year.

Leaves are the most active organs in processes such as photosynthesis, respiration and perspiration. The estimation of leaf area is of great importance for the analysis of plant growth, as it is closely related to the photosynthetic efficiency of plants [45]. Plants irrigated with magnetically treated water showed an increase in leaf growth and development, which could enhance photosynthesis (Figure 4B). Selim et al. [27] reported similar results for *Solanum lycopersicum* L. (tomato) and *Pisum sativum* L. (pea) irrigated with magnetically treated water. These results are in agreement with those obtained in leaf and stomatal areas and stomatal density estimations, as under normal conditions, stomata play a fundamental role in the water loss process in the form of steam from leaf surfaces during transpiration and stomatal opening and closure movements, which relates to the water supply of plants and conservation of plant homeostasis. This plant organ is directly involved in CO_2_ uptake and, together with chlorophyll pigments, enables good plant development and growth [46].

Plant growth does not take place uniformly; instead, it concentrates in specific and distinctive areas. Initial plant growth is essential in certain parts of the plant showing active cell division known as meristems [45]. This behavior is expressed in the same way in the growth and development of the stem, where axillary and apical shoots have meristematic tissue capable of differentiating and starting their elongation. Leaves also play a key role in plant growth, as different metabolic processes take place in leaves with the presence of minerals, growth regulators and enzymes codified by a specific gene in each type of plant.

Water plays a pivotal role in seed growth and development. Water absorption is the first process that occurs during seed germination. All hydrolysis products feed the embryo so it can start growing [47]. The biological function of water is related to the hydration of the new plant, its nutrition and the production of essential compounds necessary for the germination process, thus enhancing embryo cell elongation and radicle emergence.

These results match those obtained for other species of leguminous plants reported by Moussa [40], who found a significant increase in the crop yield and weight of *Phaseolus vulgaris* L. cv. Master. However, for the production of the seeds of *Phaseolus vulgaris* L., the type of water used for magnetic treatment, exposure times and magnetic induction must be taken into consideration [26]. Other elements to be considered include endogenous factors such as genotype and species varieties, which have a different response depending on the sowing cycle and time [42].

Chlorophylls are pigments that enable light absorption in a very effective way as they possess conjugated double bond systems, according to Azcón-Bieto and Talón [48], and enhance photosynthesis [39]. Latef et al. [49] showed that in lettuce seeds (*Lactuca sativa* var. *cabitat* L.) seedlings, they used various intensities of the static magnetic field, and the best result was obtained in the treatment group with a magnetic induction of 770 m, specifically in terms of the growth parameters and metabolism compared to the control group. Photosynthetic pigments were induced markedly in the static magnetic field group, especially the chlorophyll a pigment.

Solar energy is absorbed by the photosynthetic pigments found in plant leaf chloroplasts; chlorophyll a and b are the most abundant in plants. All chlorophylls have a complex ring chemically related to the porphyrin-like groups found in the hemoglobin in animals and cytochromes. In addition, a long hydrocarbon tail is attached to the structure of the ring, which also contains weakly bound electrons and is part of the molecule involved in electron transfer and redox reactions [50]. In this case, as the content of chlorophyll pigments in the seedlings treated with magnetized water increased, the capacity to carry out photosynthesis was enhanced, thus providing greater growth vigor and development.

Selim et al. [27] reported that magnetized water increased photosynthetic pigments in pea plants (*Pisum sativum* L.) and chickpea (*Cicer arietinum*) and *Solanum lycopersicum* L. (tomato). The results obtained for these species are similar to those reported in the literature [37], where it is stated that magnetic field intensity and time of exposure influence the concentration of photosynthetic pigments, which may increase, decrease or maintain their composition with respect to plants irrigated with unmagnetized water. In this case, a decrease in the concentration of these chemical substances (statistically significant difference with a 95% confidence level) is observed, probably as a response to magnetic field overexposure.

The biological effect of the magnetic field depends on the magnetic field intensity. Depending on the magnetic field intensity, the accumulation of chemical substances can increase, decrease or remain unchanged [51]. Moussa [40] determined the concentration of photosynthetic pigments in *Phaseolus vulgaris* L. cv. Master and reported higher values in plants irrigated with magnetically treated water. They also found that carotenoid content and photosynthetic activity increased compared to the control group of the same species.

The results reported in this study are similar to those obtained by these authors, even though they used a 30 mT magnetic induction. Their research also suggests that magnetic fields applied to irrigation water can stimulate plant defensive systems, provided that photosynthesis and the translocation of the products obtained from this process are more efficient due to the mobilization of some growth regulators and enzymes determined in their studies. The results obtained in this paper with the use of a different magnetic induction range of 100–150 mT could lead to processes similar to those already described by these authors.

Magnetic treatment applied to irrigation water has led to increased carbohydrate concentrations, thus improving the photosynthesis and nutritional value of *Phaseolus vulgaris* L. Several authors have reported increments in the content of the total available carbohydrates (monosaccharides, disaccharides, polysaccharides) of *Vicia faba* L. plants irrigated with magnetically treated water compared to plants irrigated with water without magnetic treatment [36]. The increase in the concentration of carbohydrates was possible due to the close relationship between stomatal conductance and photosynthesis; this could be the result of bioenergetic structural excitation with increased cell pumping and enzymatic stimulation. During this metabolic process, a huge variety of chemical compounds are obtained, including indole acetic acid growth regulators (IAA) and proteins, as the expression of genes that encode them has been affected [36].

These results may be related to the effect of the static magnetic field on irrigation water, which generates chemical–physical changes. Pang and Deng [25] claim that the magnetic treatment of water and its effects on the chains of hydrogen bonds forming water molecules can generate a transfer of protons from the hydrogen bonds of closed chains, which may interact with the magnetic field applied externally. At the same time, due to these magnetic interactions, closed chains can be ordered. Therefore, for the purpose of this research, this could explain why the treatment of water used for the irrigation of *Phaseolus vulgaris* L. with an induction range of 100–150 mT (0.1–0.15 T) has also undergone some changes in its solubility, surface tension, electrical conductivity and pH, leading to changes in its physical–chemical parameters. In addition, it is argued that magnetic treatment can affect the cluster composition that structures water molecules. These changes lead to a reduction in the molecular diameter and complexity; plants assimilate water more easily, and the transport of nutrients necessary for metabolism occurs more efficiently.

When the seeds of *Phaseolus vulgaris* L. are irrigated with magnetized water, they capture a signal induced by the behavior of excited water that presents in the form of physical stress at the cellular and molecular levels, causing changes in cell permeability, all of which have been reported. These interactions may have allowed soil water absorption and the transport of ions to be carried out more efficiently through the seed coat, thus starting the softening process.

The irrigation of *Phaseolus vulgaris* L. seeds with magnetically treated water under a magnetic induction of 100 and 150 mT had positive effects compared to the experimental group without magnetic treatment. The anatomical, morphological and physiological characteristics of the species, as well as the germination of its seeds, showed changes that enhance its primary and secondary metabolism. The technology employed in this study did not have negative effects on the plant nor did it affect the presence of structures or the net content of the assessed compounds. Its use in the cultivation of *Phaseolus vulgaris* L. might represent a viable alternative for the improvement of the plant and may lead to the development of state and nonstate organic farming productions.

## 4. Materials and Methods

### 4.1. Plant Material

Experiments were performed in the Plant Biotechnology Laboratory of the National Center for Applied Electromagnetism (CNEA), Santiago de Cuba, Cuba. *Phaseolus vulgaris* L. seeds were obtained from the seed laboratory attached to the Provincial Division of the Ministry of Agriculture (MINAG). During the experiments, the temperature values was in the range of 32 ± 2 °C; the average mean relative humidity was between 70 and 80%, and the rainfall was between 60 and 65 mm^3^. The Eastern Center for Ecosystems and Biodiversity (BIOECO), holder of registration identification No. 21842, was in charge of plant species classification.

### 4.2. Crop Conditions

A substrate composed of a mixture of soil and organic matter (3:1) was used. In addition, biological control of pathogens, bacteria and fungi was carried out in compliance with standards set out by the Center for Industrial Biotechnology Studies (CEBI) of the University of Oriente (Table 2).

### 4.3. Irrigation Water Characteristics

Irrigation was performed twice a day through an aerial microjet system for 30 min. The accessories used were an ITUR pump and valve-controlled distributor system that ensures irrigation is run in sections. Water tests were performed at CNEA’s LESA Lab. In the water sample analyzed, pH, total hardness and total dissolved solids values were below the maximum admissible limits (MAL) for chemical components, which may affect the organoleptic quality of drinking water (Cuban Standard 827:2012) (Table 3).

### 4.4. Description of Static Magnetic Treatment of Irrigation Water

Seeds were planted in two 12 m long and 0.5 m wide beds at CNEA’s experimental plot farm. The total sample size was 80 plants; each experimental group included 40 plants.

Treatments included two experimental groups: treatment 1 (control group): plants grown with water without magnetic treatment; treatment 2: plants grown with water magnetized with a static magnetic field (MTW) between 100 and 150 mT.

For the magnetic treatment, an external permanent magnet device designed, built and characterized at the National Center for Applied Electromagnetism was used. The device produced a magnetic induction range between 100 and 150 mT measured with a 192041 Soviet Microwebermeter (relative error less than 5%). These results were verified with nuclear magnetic resonance equipment and a 410 Gauss meter/Teslameter (relative error of 0.01 G) [52].

### 4.5. Determination of the Effect of Irrigation with Magnetically Treated Water on the Germination of Phaseolus vulgaris L. Seeds

Germination percentage: germinated seeds were counted for 24 days. Using these data, the seed germination percentage was calculated using the formula P = (seeds germinated/total seeds) × 100, according to Abdul-Baki and Andenson [53].

Hypocotyl length: measured from the base of seed hypocotyl to the starting point of epicotyl using a ruler (cm) following the methodology proposed by Abdul-Baki and Andenson [53].

Vigor index I: calculated using the formula suggested by Abdul-Baki and Andenson [53].

Vigor index I = germination percentage × hypocotyl length

### 4.6. Determination of Irrigation with Magnetically Treated Water on the Anatomical and Morphological Characteristics of Phaseolus vulgaris L. Seeds

Anatomical characters comprise stomatal density and area, whereas morphological characters include stem length, leaf area and seed area and weight. These parameters were estimated as follows:

Stomatal density and area: calculated using the stomatal impression method. The sample was taken from the underside (abaxial side) of the leaf; the epidermal impression obtained was observed through an optical microscope (Olympus, Cx41, Japan) under 10× and 40× magnification. The number of stomata was calculated in three fields, and stomatal length and width were determined according to Ortega and Rodés [54]. The results were expressed as the number of stomata (µm^2^), with three replications.

Stomatal area: Stomal length and width were measured and multiplied by π = 3.14, as suggested by Ortega and Rodés [54]. Values were expressed in µm^2^ with three replications.

Stem length: The length of the stem was measured using a measuring tape (cm) in 40 plants of each experimental group from the base of the stem to the main root apex.

Length of leaf area: Leaf area was measured in the same plants of each experimental group. The following equation was used: LA= leaf length × leaf width × π (π = 3.14).

Seed length and width: The length and width of 100 seeds from each experimental group were measured using a calibrated ruler in centimeters (cm).

Seed weight: Seed weight was estimated with a Sartorious^®®^ digital analytical balance (BS 124S, China; accuracy: 0.1 mg). Values were expressed in grams (g) with three replications for each experimental group.

### 4.7. Determination of the Effect of Irrigation with Magnetically Treated Water on the Physiological Characteristics of Phaseolus vulgaris L. Seeds

#### 4.7.1. Concentration of Photosynthetic Pigments

To determine the concentration of photosynthetic pigments, 1 g of fresh leaf was weighed. Extracts were prepared with 50 mL of solvent (96% ethanol) for both the plant group irrigated with MTW and the control plant group. Leaves were macerated in a porcelain mortar with said solvent. They were filtered using filter paper, and ethanol was added to a volume of 50 mL; 5 mL of this solution was transferred to a 50 mL volumetric flask and brought up to the mark with 96% ethanol. Absorbance peaks of extracts were determined using a Genesys spectrophotometer (10 UV, United States) according to Ortega and Rodés [54]. Absorbance was obtained at wavelengths of 440, 472, 643, 645, 649, 654, 663 and 665 nm. The formulas shown below were employed:

Ca = 0.0127 A668 − 0.00269 A648

Cb = 0.0229 A665 − 0.00468 A663

Ccarot = 4.695 A440.5 − 0.268 Ca + b

Values were expressed in g mL^−1^ with three repetitions.

#### 4.7.2. Concentration of Carbohydrates

Estimation of total carbohydrates was carried out using the phenol-sulfuric colorimetric method described by Dubois et al. [55]. One milliliter of *Phaseolus vulgaris* L. was taken from the experimental groups. For the preparation of the glucose standard solution, 50 mg of glucose was diluted in 100 mL of distilled H_2_O as a final volume. Absorbance was determined at 492 nm using a Genesys spectrophotometer (10 UV, United States). The results were expressed in D-glucose from a calibration curve between 0.6 and 1.6 mgmL^−1^ obtained for this compound. The total carbohydrate content was expressed in mg of saccharides g^−1^ of dry weight. Distilled water was used as a blank in all three replicates. The mathematical equation used to determine the sample concentration was the following: “Extract Conc. = D-glucose Equivalent Conc.de * dilution math conc”.

### 4.8. Statistical Analysis

A completely randomized experimental design with three replications was used. The Kolmogorov–Smirnov test was performed to check if the data had a normal distribution and verify homogeneity of variances. Descriptive statistics and simple classification analysis of variance (ANOVA) were performed. Student’s t-test was used to compare two samples. All statistical analyses were performed with a significance value of *p* < 0.05 using Statgraphics Centurium XV for Windows (Graphics Software Systems, STCC, 2000, Inc., Rockville, MA, USA), Basic Statistics, Prisma 5.01 and Origen 6.0.

## 5. Conclusions

The magnetically treated water under a magnetic induction of 100 to 150 mT stimulated the germination, growth and biomass production of the bean plants. This positive impact of magnetically treated water on beans was associated with an increase in stomatal (38%) and leaf area (109%), together with stem length (35%), vigor index (100%), leaf area (109%) and seed weight (16%), as well as the anatomical (chlorophyll a and b, with 13 and 21%, respectively) and carbohydrates (26%). These results can be translated as a greater seed recovery and better crop performance for food purposes. Thus, the consumption of beans treated as a food with a high nutritional value is possible. The data also demonstrate that no malformations or abnormal changes were detected in plants grown with magnetically treated water, so this technology is a commercially successful agricultural practice, and magnetic treatment can be used as a suitable environmentally friendly physical method capable of contributing to sustainable agriculture.

## Figures and Tables

**Table 1:** Mean values of hypocotyl length and vigor index of *Phaseolus vulgaris* L. grown with magnetically treated water.

Experimental Group ^1^	Hypocotyl Length (cm)	Vigor Index (%)
Control	1.28 ± 0.36	66.77 ± 18.44
MTW	1.78 ± 0.58 *	133.79 ± 43.70 *

^1^ Control: plants grown without magnetically treated water, MTW: plants grown with magnetically treated water under a static magnetic field between 100 and 160 mT. * indicates statistically significant differences (*p* < 0.05).

**Table 2:** Physico-chemical analysis of soil in CNEAʹs experimental plot farm for the cultivation of *Phaseolus vulgaris* L.

Characteristics	Mean Values	Characteristics	Mean Values
Electrical conductivity (µScm^−1^)	397	Aluminum (%)	12.67
Potassium oxide (%)	0.30	Magnesium oxide (%)	8.06
Phosphorous (%)	0.940	Cobalt (%)	˂0.005
Calcium oxide (%)	7.29	pH	7.84
Iron (%)	3.9	Manganese (%)	0.20

**Table 3:** Physico-chemical analysis of irrigation water at CNEA’s experimental plot farm for the cultivation of *Phaseolus vulgaris* L. *: expressed as CaCO_3_.

Parameters	Control	MTW
Total hardness * (mg L−1)	129.28 ± 0.00	162.95 ± 2.33
Calcium hardness * (mg L−1)	100.80 ± 0.00	92.74 ± 0.00
Magnesium hardness * (mg L−1)	28.48 ± 0.00	70.21 ± 2.33
Phenolphthalein alkalinity * (mg L−1)	0.00 ± 0.00	0.00 ± 0.00
Total alkalinity * (mg L−1)	149.18 ± 0.00	145.15 ± 0.00
Bicarbonate * (mg L−1)	149.18 ± 0.00	145.15 ± 0.00
Chlorides (mgL−1)	23.24 ± 0.54	24.38 ± 1.08
pH	7.71 ± 0.01	7.70 ± 0.00
Electrical conductivity µScm−1	301.00 ± 2.00	302.00 ± 0.00
Total dissolved solids (mg L−1)	150.53 ± 0.97	151.00 ± 0.00
Salinity (ppt)	0.15 ± 0.00	0.15 ± 0.00
Water speed (ms^−1^) Pump flow m^3^ h^−1^	1.4–1.6 2.54–2.91	

## Data Availability

Not applicable.
